# Identification of a compound heterozygous missense mutation in *LAMA2* gene from a patient with merosin‐deficient congenital muscular dystrophy type 1A

**DOI:** 10.1002/jcla.23930

**Published:** 2021-09-16

**Authors:** Afshin Khorrami, Pouya Goleij, Vahidreza Karamad, Elham Taheri, Behrouz Shadman, Parisa Emami, Gholamreza Jahangirzadeh, Saba Hajazimian, Alireza Isazadeh, Behzad Baradaran, Mansour Heidari

**Affiliations:** ^1^ Young Researchers and Elit Club Varamin‐Pishva Branch Islamic Azad University Varamin‐Pishva Iran; ^2^ Department of Genetics Faculty of Biology Sana Institute of Higher Education Sari Iran; ^3^ Department of Medical Biology Faculty of Medicine Ege University Izmir Turkey; ^4^ Department of Pharmaceutical Biotechnology Tabriz University of Medical Sciences Tabriz Iran; ^5^ Department of Genetics Ahar Branch Islamic Azad University Ahar Iran; ^6^ Department of Genetics Tabriz Branch Islamic Azad University Tabriz Iran; ^7^ Immunology Research Center Tabriz University of Medical Sciences Tabriz Iran; ^8^ Department of Medical Genetics Tehran University of Medical Sciences (TUMS) Tehran Iran

**Keywords:** congenital muscular dystrophy, *LAMA2* gene, mutation, whole‐exome sequencing

## Abstract

**Background:**

Merosin‐deficient congenital muscular dystrophy type 1A (MDC1A) is occurred by mutations in *LAMA2* gene that encodes the laminin α2 chain (merosin). MDC1A is a predominant subtype of congenital muscular dystrophy. Herein, we identified two missense mutations in *LAMA2* gene in compound heterozygous status in an Iranian patient with MDC1A using whole‐exome sequencing (WES).

**Methods:**

In the present study, we evaluated genetic alterations in an Iranian 35‐month‐old boy with MDC1A and his healthy family using WES method. The identified mutations further confirmed by Sanger sequencing method. Finally, *in silico* analysis was conducted to further evaluation of molecular function of the identified genetic variants.

**Results:**

We identified two potentially pathogenic missense mutations in compound heterozygous state (c.7681G>A p.Gly2561Ser and c.4840A>G p.Asn1614Asp) in *LAMA2* gene as contributing to the MDC1A phenotype. The healthy parents of our proband are single heterozygous for identified mutations. These variants were found to be pathogenic by *in silico* analysis.

**Conclusions:**

In general, we successfully identified *LAMA2* gene mutations in an Iranian patient with MDC1A using WES. The identified mutations in *LAMA2* gene can be useful in genetic counseling, prenatal diagnosis, and predicting prognosis of MDC1A.

## Graphical abstract

Genetic alterations in an Iranian 35‐month‐old boy with MDC1A and his family were investigated using WES method. Sanger sequencing method was used in order to confirmation of the identified mutations. We identified two pathogenic missense mutations in the compound heterozygous state on the *LAMA2* gene as contributing to the MDC1A phenotype.

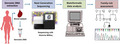

## 1 INTRODUCTION

**FIGURE 1:**
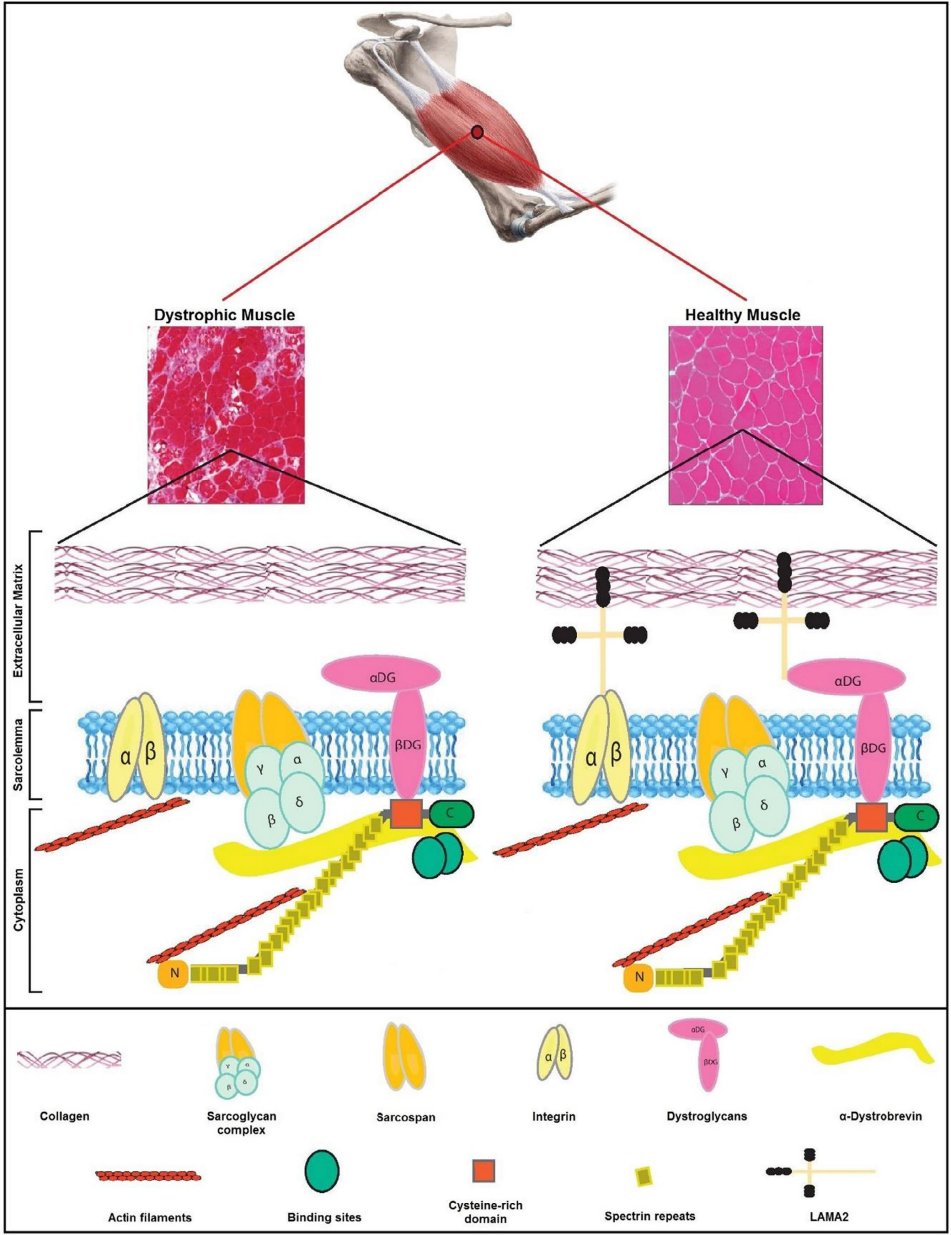
The major proteins involved in congenital muscular dystrophies: location and interaction

The merosin‐deficient congenital muscular dystrophy type 1A (MDC1A) with autosomal recessive inheritance affects the peripheral and central nervous system in children.^1^ This disorder is characterized by increased levels of creatine kinase (CK) in serum, hypotonia, abnormalities of white matter, poor cry and suck, failure to thrive, and muscle weakness.^2^, ^3^ The prevalence of MDC1A is 1–9 per 1,000,000 children and constitutes 1–6% of all congenital muscular dystrophy cases.^4^, ^5^ Furthermore, this disorder is rarer in Asian population and more common in European countries and Caucasians race.^5^, ^6^ The various mutations in the *LAMA2* gene (with 65 exons) are the main cause of MDC1A.^7^ Other major factors involved in congenital muscular dystrophies are presented in Figure 1.

*LAMA2* gene, on chromosome 6q22, encodes the laminin‐α2 chain, which connects with laminin‐γ1 and laminin‐β1 chains and forms the heterotrimeric laminin‐211 protein. The laminin‐211 protein is a main component of the extracellular matrix and the skeletal muscle membrane.^8^, ^9^ The interaction of this protein with various matrix macromolecules plays an important role in tissue phenotypes, cell movement, and cell differentiation.^9^ Previous studies reported that the genetic variations of MDC1A are compound heterozygous or homozygous mutations.^6^, ^10^ Moreover, *de novo* mutations are the rare events and a few have been reported in MDC1A patients.^11^, ^12^

The early diagnosis of MDC1A is based on high serum concentrations of CK, deficiency of merosin in skin or muscle biopsy, alterations in white matter on brain, and clinical examination.^11^ Previous studies reported the efficiency of whole‐exome sequencing (WES) for the molecular diagnosis of the congenital muscular dystrophy.^13^, ^14^ However, use of WES method is not cost‐effective in patients with clinical overlap. A previous study on an Iranian patient with congenital muscular dystrophy revealed an improved diagnostic yield of WES method.^15^

In the present study, potentially pathogenic mutations of *LAMA2* gene were evaluated in an Iranian patient with MDC1A using WES along with Sanger sequencing. We identified two mutations in the compound heterozygous state on *LAMA2* gene. Furthermore, *in silico* analysis suggests that these mutations can cause production of a defective protein by *LAMA2* gene.

## 2 MATERIALS AND METHODS

### 2.1 Case presentation

**FIGURE 2:**
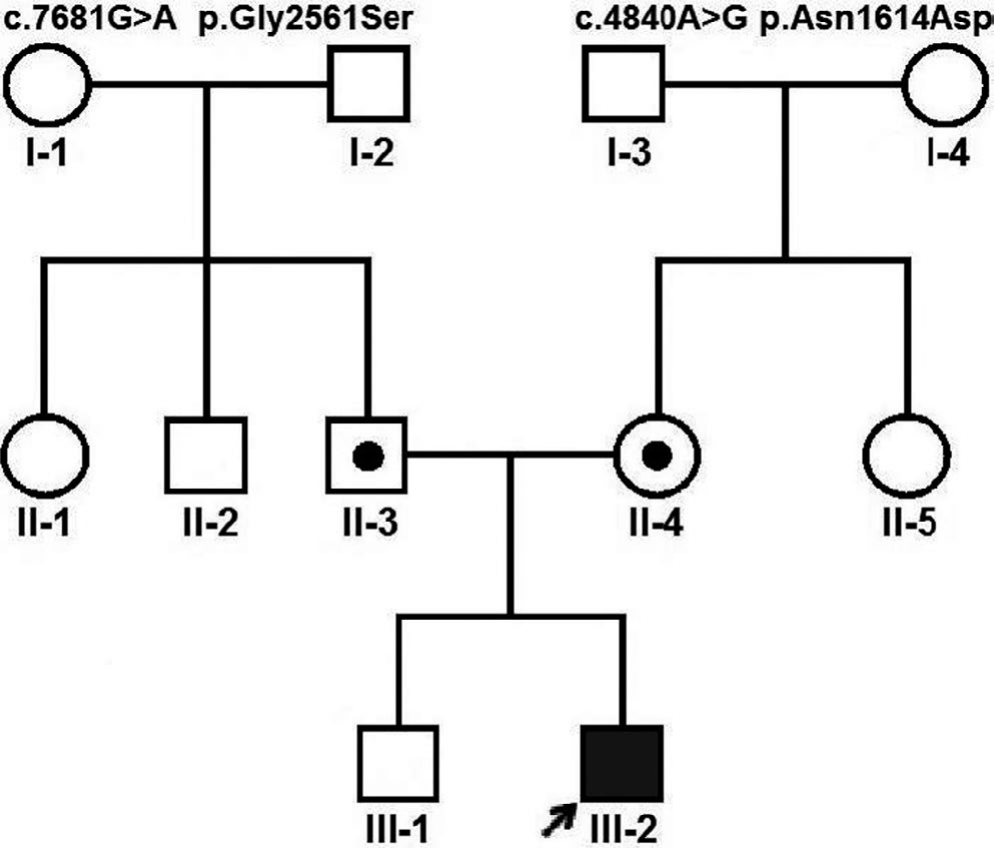
The pedigree analysis of an Iranian family with *LAMA2* gene mutation. Both parents are single heterozygous, and affected patient is in compound heterozygous condition

This patient is a 35‐month‐old male referred Aria Gene Medical Genetics Laboratory, Qom, Iran. He was the second child in a healthy family without any neuromuscular diseases history. This patient is the offspring of a non‐consanguineous marriage. His only older brother is healthy without any problems (Figure 2). He was born normally at 36rd weeks of pregnancy through a spontaneous vaginal delivery. He did not show any abnormalities in neonatal period and was discharged from the hospital on third day. He was breastfeeding, and he had no problems for the first year of his life. After one year, physical developmental and motor milestones delays were observed (sat at 10 months, crawled at 16 months, stood unaided at 25 months, and started walking at 31 months). His family worries started at 25 months, because he cannot stand unsupported and always had difficulty running. The preliminary examinations revealed a tightness of ankles and mild proximal muscle weakness. Moreover, this patient was with bilateral clubfoot, cataract, vermis hypoplasia, and microphthalmia. However, development of the speech and intellectual was normal. There was no vision or hearing problems. According to the ethical standards of Helsinki Declaration, the studied patient and his parents were informed about the aim of present study and signed an informed consent. The present study was approved by the Institutional Review Board (IRB), Qom University of Medical Sciences, Qom, Iran.

### 2.2 Genomic DNA extraction

The peripheral blood lymphocytes (5 ml) were received from the studied patient and his healthy parents. Extraction of the genomic DNA was conducted using a standard DNA purification kit (Roche, Switzerland). The purity and quantity of the genomic DNA samples were evaluated using NanoDrop instrument (Thermos Fisher Scientific, USA). The genomic DNA samples with appropriate OD 260/280 ratio (1.7 to 1.9) were further evaluated for quality. The quality of the genomic DNA samples was evaluated using electrophoresis on 1% agarose gel. Finally, the genomic DNA samples without smear or diffuse and with a sharp band were stored at −20℃ and then used for molecular analysis.^16^

### 2.3 Whole‐exome sequencing (WES)

The WES was used for the proband, and his healthy mother and father. The capture of the exome sequence was conducted using the SureSelect Human All Exon V5 Kit (Agilent Technologies, United States). The capture library was sequenced via 2×150 paired‐end sequencing on a Hiseq2000 Sequencer (Illumina, United States).^17^

### 2.4 Analysis of sequencing data

The sequence reads were aligned to human reference genome by Burrows‐Wheeler Aligner algorithm, and then, processing was performed using SAMtools. All small deletions–insertions (indels) and single nucleotide polymorphisms (SNPs) were analyzed using Genome Analysis Toolkit (GATK) and VarScan software. The variants annotate was conducted using the ANNOVAR software. The variants in homozygous condition were excluded, and frameshift, missense, and nonsense mutations were considered as pathogenic. The pathogenic potential of the missense mutations was analyzed using the MutationTaster, FATHMM, Polyphen‐2, M‐CAP, PROVEAN, SIFT, REVEL, MetaLR, and MetaSVM software. All variants with autosomal recessive, dominant, and X‐linked inheritance models were assumed for the analysis. The mutations passed these filtering were considered as pathogenic.^18^

### 2.5 Sanger sequencing

The Sanger sequencing was performed to validate the candidate mutations in proband and his parents. The target exons containing mutations of *LAMA2* gene were amplified using polymerase chain reaction (PCR) and designed primers. The products of PCR were sequenced using ABI 3130 automated sequencer (Applied Biosystems, Forster City, CA, USA). The obtained sequences were analyzed using Mutation Surveyor software.^19^

## 3 RESULTS

### 3.1 Clinical findings

In this study, an Iranian family member with congenital muscular dystrophy was evaluated. The clinical experiments were all normal for gland function, renal function, hepatic function, lipoproteins, triglyceride, cholesterol, glucose, alkaline phosphatase, electrolyte, thyroid, ammonia, lactic acid. The karyotype analysis was normal in the proband. However, CK level was at 812 IU/l (normal <200 IU/l). The electromyography revealed a myopathic process. The magnetic resonance imaging (MRI) or brain revealed an agyria area in occipital cortex. The T2‐weighted images were detected swelling and widening of gyri, extensive white matter abnormalities, mainly frontal. The cardiac function has decreased and the dilated cardiomyopathy with dysfunction of left ventricle contractility was detected. Therefore, we suggested MDC1A as a possible diagnosis (Table 1).

**TABLE 1:** The clinical features of the studied patient with MDC1A

No.	Clinical features	Characteristic	No.	Clinical features	Characteristic
1	Age of onset	Birth	14	Gland function	Normal
2	Consanguineous marriage	Yes	15	Renal function	Normal
3	Karyotype analysis	Normal	16	Hepatic function	Normal
4	Current age (month)	35	17	Lipoproteins	Normal
5	Serum CK	812 IU/l	18	Triglyceride	Normal
6	Max. motor milestone	Sat unsupported	19	Cholesterol	Normal
7	Contractures	Yes	20	Glucose	Normal
8	Mental Retardation	No	21	Alkaline phosphatase	Normal
9	White Matter Changes	Yes	22	Electrolyte	Normal
10	Eye involvement	Myopia	23	Thyroid	Normal
11	Cardiac function	Mild hypertrophy	24	Ammonia	Normal
12	Scoliosis	No	25	Lactic acid	Normal
13	Facial dysmorphism	No	26	Respiratory function	Normal

### 3.2 Detection of LAMA2 mutation using WES

The obtained results of WES revealed a heterozygous missense mutation c.7681G>A p. Gly2561Ser (exon 55) in the *LAMA2* gene. Moreover, another heterozygous missense mutation c.4840A>G p. Asn1614Asp (exon 33) was detected in the *LAMA2* gene. These indicated that the compound heterozygous variants (c.7681G>A and c.4840A>G) co‐segregated with this disease in this family.

### 3.3 Confirmation of detected LAMA2 mutation using Sanger sequencing

The two identified mutations (c.7681G>A and c.4840A>G) in the *LAMA2* gene were confirmed using Sanger sequencing. We found that the two mutations of *LAMA2* gene were in the compound heterozygous state in the studied patient. However, parents of the proband were in heterozygous state for c.4840A>G (mother) and c.7681G>A (father) mutations.

## 4 DISCUSSION

MDC1A is an autosomal recessive disease which occur by mutations in *LAMA2* gene and represents the predominant subtype of congenital muscular dystrophy.^4^ Important presentations of MDC1A are increased white matter abnormalities, increased levels of CK, and absence of laminin‐α2 chain around muscle fibers.^20^ However, due to genetic and clinical heterogeneity, precise molecular diagnosis of MDC1A is still a challenge for clinicians. Recently, targeted WES method has emerged as a powerful molecular diagnosis tool which widely used to identify causal genes in genetic diseases.^21^

In this study, we used WES method combined with Sanger sequencing to identify genetic causes of MDC1A in an Iranian patient. Our study identified two mutations in *LAMA2* gene in an Iranian patient with MDC1A in compound heterozygous status. Further *in silico* analysis demonstrated that these mutations are possible pathogenic in our proband. Other family members with heterozygous mutations of *LAMA2* gene (c.7681G>A or c.4840A>G) were healthy, which may be due to reserving a partly normal *LAMA2* gene‐encoded protein. This evidence demonstrated that identified compound heterozygous mutations were the cause of MDC1A phenotype in our proband.

Previously, diagnosis of MDC1A was performed according to the clinical presentations, such as white matter alternations, high levels of serum CK, severe congenital hypotonia, and deficiency of merosin expression in biopsied muscle.^4^ The muscle biopsy seems to be an essential method to confirm the diagnosis of congenital muscular dystrophy. However, the molecular genetic diagnosis may be an alternative method if the clinical phenotypes support the diagnosis of congenital muscular dystrophy.^22^ Our proband was suspected to have congenital muscular dystrophy due to white matter abnormalities, high serum CK levels, and appendicular hypotonia. Therefore, we used molecular genetic analysis to identification of possible mutations of *LAMA2* gene which is responsible for the symptoms of MDC1A.

Interaction of merosin with various matrix macromolecules and skeletal muscle membrane plays an important role in tissue phenotypes, cell movement, and cell differentiation. To date, approximately 90 mutations have been described in *LAMA2* gene.^23^ In present patient, we identified two missense mutations in heterozygous status which is located in exons 33 and 55. The G domain at the C terminus of merosin (exons 46–64) is responsible in the connection between the dystrophin‐glycoprotein and the extracellular matrix.^24^ Deficiency of this domain disrupts the link between subsarcolemmal cytoskeleton and extracellular matrix, which causes muscle degeneration.^1^ The severe phenotype of our proband may explain by this evidence.

Limited evidence has been reported for cardiac defects related to the laminin‐a2 deficiency in patients with MDC1A. A previous study specifically addressed involvement of the cardiac defects in patients with laminin‐a2 deficiency.^25^ The reported cardiac abnormalities in patients with MDC1A included borderline changes in cardiac function, dilated cardiomyopathy, and right bundle branch block. Moreover, cerebral white matter abnormalities are commonly reported in patients with MDC1A.^20^ However, underlying mechanisms responsible for white matter abnormalities in patients with MDC1A remain elusive and thus cause abnormal signal intensity of white matter.^26^ In our proband, we observed white matter abnormalities of parietal and occipital lobes, whereas the corpus arenaceum and cerebellum were normal. The white matter abnormalities are a typical feature of patients with MDC1A compared with other congenital muscular dystrophy subtypes.

Generally, we identified two potentially pathogenic mutations in a compound heterozygous state (c.7681G>A p. Gly2561Ser and c.4840A>G p. Asn1614Asp) in *LAMA2* gene responsible for MDC1A phenotype in an Iranian patient. These results can refine prenatal diagnosis, genetic counseling, and treatments of patients with *LAMA2* gene‐caused MDC1A.

## CONFLICT OF INTEREST

The authors declare that they have no competing interests.

## ETHICAL APPROVAL

All procedures performed in the studies involving human participants were in accordance with the ethical standards of the Institutional Review Board of Qom University of Medical Sciences and with the 1964 Helsinki Declaration and its later amendments or comparable ethical standards. Informed consent was obtained from patient and parents.

## Data Availability

The raw data analyzed during the current study are not publicly available due to the aim to protect the confidentiality of the patients but are available from the corresponding author on reasonable request.
